# Aminooxyacetic acid (AOAA) sensitizes colon cancer cells to oxaliplatin via exaggerating apoptosis induced by ROS

**DOI:** 10.7150/jca.35375

**Published:** 2020-01-20

**Authors:** Taohua Yue, Shuai Zuo, Dingfang Bu, Jing Zhu, Shanwen Chen, Yongchen Ma, Ju Ma, Shihao Guo, Long Wen, Xiaoqian Zhang, Jianwen Hu, Yurong Wang, Zihao Yao, Guowei Chen, Xin Wang, Yisheng Pan, Pengyuan Wang, Yucun Liu

**Affiliations:** 1 Division of General Surgery, Peking University First Hospital, Peking University, 8 Xi Shiku Street, Beijing, 100034, People's Republic of China.; 2 Central laboratory, Peking University First Hospital, Peking University, 8 Xi Shiku Street, Beijing, 100034, People's Republic of China.

**Keywords:** colorectal cancer, oxaliplatin, hydrogen sulfide, AOAA, reactive oxygen species, apoptosis.

## Abstract

**Background:** As the third confirmed gaseous transmitter, the role of hydrogen sulfide (H_2_S) in the pathogenesis of multiple types of cancer has been attracting increasing attention. Increased expression of cystathionine β-synthase (CBS) and H_2_S in colon cancer tissue samples has been validated and tumor-derived H_2_S, mainly produced by CBS, stimulates bioenergetics, cell proliferation, and angiogenesis in colon cancer. Recently, the therapeutic manipulation of H_2_S has been proposed as a promising anticancer approach. However, the effect of aminooxyacetic acid (AOAA), which has been widely used as an inhibitor of CBS dependent synthesis of H_2_S, on the chemotherapeutic effect of oxaliplatin (OXA) and the underlying mechanisms remain to be illustrated.

**Methods:** We examined the expression of CBS in human colorectal cancer specimens and matched normal mucosa by immunohistochemistry. The effect of AOAA on the sensitivity of colon cancer cells to OXA and the level of apoptosis induced by caspase cascade was investigated in both HCT116 and HT29 cell lines utilizing CCK-8 assays, flow cytometry analysis and western blot analysis. The endogenous levels of reactive oxygen species (ROS) were detected fluorescently by DCF-DA, and glutathione (GSH) levels were measured by a Total GSH Detection Kit. Tumor bearing xenograft mouse models and *in vivo* imaging systems were further used to investigate the effect of AOAA* in vivo* and immunohistochemistry (IHC) and TUNEL analysis were performed.

**Results:** In the current study, we confirmed CBS, the main target of AOAA, is overexpressed in human colorectal cancer by immunohistochemistry. The inhibitory effect of AOAA on the synthesis of H_2_S was validated utilizing fluorescent probe and specific electrode. AOAA significantly reduced the IC50 values of OXA in both colon cancer cell lines. Co-incubation with AOAA elicited increased apoptosis induced by OXA, featured by increased activation of caspase cascade. Besides, AOAA further increased the levels of ROS induced by OXA and attenuated the synthesis of glutathione (GSH), which is a vital antioxidant. Besides, the results of *in vivo* imaging and following IHC and TUNEL analysis were in accordance with cellular experiments, indicating that AOAA sensitizes colon cancer cells to OXA via exaggerating intrinsic apoptosis.

**Conclusion:** The results suggested that CBS is overexpressed in colorectal cancer tissues and AOAA sensitizes colon cancer cells to OXA via exaggerating apoptosis both *in vitro* and *in vivo*. Decreasing the endogenous level of GSH and consequently impaired detoxification of ROS might be one of the mechanisms underlying the effect of AOAA.

## 1. Introduction

Colorectal cancer (CRC) is the third most common diagnosed cancer worldwide and the second leading cause of cancer related mortality. More than half of the patients with CRC will eventually die of this dreadful disease 1. As the standard chemotherapy regimens, FOLFOX and XELOX still play an irreplaceable role in the treatment of CRC in advanced stages. Oxaliplatin (OXA), a third-generation platinum drug, is a DNA-interacting agent that disrupts DNA replication and transcription and induces ROS production, promoting apoptosis 2,3. Although OXA improves the objective response and median overall survival rates, the main dose-limiting toxicity, peripheral sensory neuropathy and drug resistance still limit its widespread use. Thus, constant efforts need to be made to improve efficacies and attenuate dose-related side effects of OXA.

As the third confirmed gaseous signaling molecule after nitric oxide (NO) and carbon monoxide (CO), H_2_S has received increasing attention as an endogenous biological mediator in recent years 4-9. In mammalian cells, H_2_S is produced mainly by three key enzymes: cystathionine β-synthase (CBS), cystathionine γ-lyase (CSE) and 3-mercaptopyruvate thiotransferase (3-MST) 4. Like the other two gaseous transmitters, NO and CO, the physiological action of H_2_S is also bidirectional: a low concentration of H_2_S exerts cytoprotective effects, and a high concentration of H_2_S shows cytotoxic effects 10. Abnormal H_2_S metabolism is implicated in multiple types of cancers 11, 12. Increased expression of CBS and H_2_S has been validated in colon cancer tissue samples and cell lines. Silencing CBS or inhibiting its enzymatic activity significantly attenuated the proliferation of colon cancer cells both *in vitro* and* in vivo* 13. Similar effect has been validated in ovarian cancer, breast cancer and lung adenocarcinoma, indicating that the CBS/H_2_S axis plays an essential role in the pathogenies of multiple types of cancer 14-16.

However, the effect of inhibition of the CBS/H_2_S axis on the sensitivity of colon cancer cells to OXA has not been illustrated. Increased production of ROS and P53 has been implicated in the apoptosis of colon cancer cells induced by OXA 3, 17-21. One of the mechanisms underlying the acquired resistance of OXA is increased intracellular GSH and consequently increased detoxification of ROS 22. While, the CBS inhibitor aminooxyacetic acid (AOAA) is also involved in regulating ROS levels and P53 activation 12, 16.Considering the antioxidant effect of H_2_S and the pivotal role of increased ROS in mediating the apoptosis induced by OXA, inhibition of H_2_S synthesis might sensitize colon cancer cells to OXA. Thus, we set out to investigate the effect of AOAA on the chemotherapeutic effect of OXA in colon cancer cells both *in vitro* and* in vivo*. The results suggested that AOAA sensitized colon cancer cells to OXA via exaggerating intrinsic apoptosis. Decreasing the endogenous level of GSH and consequently impaired detoxification of ROS might be one of the mechanisms underlying the effect of AOAA.

## 2. Materials and methods

### 2.1. Cells, chemicals and reagents

Human colon cancer cell lines HCT116, DLD-1and HT29 and the nonmalignant colonic epithelial cell line NCM460 were obtained from the ATCC (Manassas, VA, USA) and cultured in McCoy's 5A medium (modified), DMEM and RPMI-1640 medium supplemented with 10% FBS and 1% penicillin-streptomycin, respectively. All cells were cultured in the optimal environment of 37 °C with 5% CO_2_. Cells treated with DMSO or PBS were used as the negative control (Ctrl). Oxaliplatin, D-Luciferin sodium, DCF-DA, Hoechst 33342 (HO), the Annexin V-FITC PI Apoptosis Detection Kit, the *In Situ* Cell Death Detection Kit, and the Cell Counting Kit-8 (CCK-8) were all purchased from Sigma-Aldrich (St. Louis, MO, USA). All antibodies were purchased from Cell Signaling Technology (Beijing, China). A new H_2_S-specific near-infrared fluorescence enhanced probe was donated by Beijing University of Chemical Technology. A Total GSH Detection Kit was purchased from Beyotime Biotechnologies (Jiangsu, China).

### 2.2. Measurement of cell viability

The CCK-8 assay was used to detect cell viability according to the manufacturer's instructions. Briefly, HCT116 and HT29 cells were cultured until ~80% confluence. HCT116 and HT29 cells were digested completely and added to each well (6,000 cells/well) of a 96-well plate (Corning, USA). According to the protocol provided by the manufacturer, at the end of treatments, add 10% CCK-8 solution to each well of the 96-well plate. Be careful not to introduce bubbles to the wells, since they interfere with the O.D. reading. To obtain a concentration of AOAA that inhibited cellular H_2_S synthesis but was noncytotoxic to cell survival, cells were treated with gradient concentrations of AOAA for 48 hours. After determining the AOAA concentrations, cells were treated with gradient concentrations of OXA in the presence or absence of this specific concentration of AOAA for 48 hours, and the IC50 values of OXA were measured.

### 2.3. H_2_S detection

To determine the inhibitory efficacy of AOAA on cellular H_2_S synthesis, the probe and a Mettler sulfur ion electrode were applied according to the manufacturer's instructions. For the qualitative detection of endogenous H_2_S, cells were seeded in a glass-bottom 35 mm plate (~ 2×10^4^ cells per well) (Corning, USA) and first incubated with DMSO or AOAA for 30 minutes, replaced with medium containing the H_2_S probe (10 µmol/L) for an additional 30 minutes and then washed with PBS twice before fluorescence imaging 23. To quantify the level of H_2_S, we measured the H_2_S content in the supernatants of HCT116 and HT29 cells treated with DMSO and AOAA for 48 hours by the electrode.

### 2.4. Flow cytometry analysis of apoptosis

An Annexin V-PI Staining Kit was applied to detect the apoptosis of HCT116 and HT29 cells treated with DMSO, AOAA, OXA and AOAA+OXA. Apoptotic cells were examined by flow cytometry according to the manufacturer's instructions (BD Bioscience, USA). The results were presented as the percentage of total cells and were compared to the proportion of four groups of apoptotic cells (early apoptosis + late apoptosis).

### 2.5. Western blot analysis

The group division was the same as that used in the cell viability and apoptosis assay. Total proteins were separated by 4-12% SurePAGE and transferred onto a PVDF membrane. After blocking in 5% BSA for 1 hour, the bands were incubated with the primary antibodies overnight at 4 °C, followed by incubation with the corresponding secondary antibodies for 1 hour. The membranes were washed with TBST after incubation with each antibody. The specific primary antibodies were used as follows: PARP (1:1000 Dilution; CST, MA, USA), cleaved PARP ( 1:1000 dilution, CST, MA, USA), P53 (1:1000 dilution, CST, MA, USA), cleaved caspase 3 (1:1000 dilution, CST, MA, USA), caspase 9 (1:1000 dilution, CST, MA, USA), Bcl-2 (1:1000 dilution, CST, MA, USA), Bax (1:1000 dilution, CST, MA, USA) and GAPDH (1:1000, CST, MA, USA). GAPDH served as the internal controls. The bands were detected using the Syngene GeneGenius gel imaging system (Syngene, Cambridge, UK). The results were analyzed by ImageJ software.

### 2.6. Measurement of intracellular ROS

To explore the mechanisms of OXA combined with AOAA in exaggerating apoptosis, a 96-well plate was seeded at 6,000 cells per well. After 24 hours of adherence, five treatments were administered: DMSO, DMSO, AOAA, OXA, and AOAA +OXA. The drug is updated once every 24 hours for a total of 72 hours. Then, either of the DMSO groups was treated with H_2_O_2_ (30 mmol/L) for 30 minutes as a positive control. Thereafter, all groups were treated with a mixture of DCF-DA (30 µmol/L) and HO (2.5 µg/ml) for 30 minutes to detect ROS and the corresponding number of viable cells, respectively. After discarding the supernatant and PBS rinse, fluorescence intensity was measured by a microplate reader, and the corresponding excitation/emission wavelengths were 490/530 nm (DCF-DA) and 340/425 nm (HO). The amount of ROS depended on the ratio of DCF-DA/HO signals per well 18.

### 2.7. Measurement of total intracellular GSH

Reports have indicated that AOAA reduces intracellular cystathionine, a precursor of GSH, thereby decreasing the antioxidant capacity of cells 16. To determine whether the intracellular increase in ROS was caused by a decrease in the antioxidant GSH, both HCT116 and HT29 cells were treated with DMSO and AOAA. Total GSH detection assays were performed using the Total GSH Detection Kit according to the manufacturer's protocol 24, 25.

### 2.8. Establishment of a mouse xenograft model of a human colon cancer cell line stably expressing luciferase

All animal studies were approved by the Institutional Animal Care and Use Committee (IACUC) of Peking University First Hospital. Athymic nude BALB/c male mice (4 weeks old) purchased from Beijing Vital River Laboratory Animal Technology Co., Ltd., were injected subcutaneously in the right armpit with 8× 10^5^ cells in 100 µl PBS. four to five days later, mice were randomly assigned to one of four groups and subjected to intraperitoneal injection : control (PBS), AOAA (9 mg/kg in PBS, 5 days per week), OXA (5 mg/kg in PBS, once a week), and AOAA + OXA (as before) 13, 14. D-Luciferin sodium was intraperitoneal administration once every two weeks for* in vivo* imaging of nude mice (500 nmol/g body weight D-luciferin for an average-sized mouse) 26. After 28 days, mice were euthanized, and xenografts were harvested and weighed.

### 2.9. Immunohistochemistry (IHC) and TUNEL assay

To measuring the proliferation of *in vivo* tumor cells, we detected two biomarkers at the protein level by IHC staining. Briefly, xenografts embedded in paraffin were cut into 4-5 µm sections and fixed onto slides. Then, the slides were deparaffinized, hydrated and microwave treated for antigen retrieval. Antigen detection and the dilution ratios were as follows: (1) Ki67 at a dilution of 1:400 (CST, MA, USA) and (2) PCNA at a dilution of 1:8,000 (CST, MA, USA). Both antibodies were incubated overnight in the dark. The next day, the corresponding secondary antibody, horseradish peroxidase (HRP)-conjugated goat anti-rabbit IgG (ZSGB-BIO, Beijing, China) was incubated at 37 °C for 30 minutes. Then, the DAB display color was as follows: Ki67, 40 seconds, and PCNA, 1 minutes. Finally, the slides were counterstained with hematoxylin, subjected to gradient alcohol and xylene dehydration, sealed with neutral gum, and counted under a microscope (Olympus, Japan) at a magnification of 20X.

To confirm that the study is clinically relevant, we searched the hospital case management system, and 11 cases of colorectal cancer patients in our hospital recent surgical resection specimens and matched normal mucosa were included in this study. Immunohistochemistry was performed to detect the expression of CBS, the main target of AOAA. Antigen detection and the dilution ratios were 1:100 (CST, MA, USA). The DAB display color was 6 minutes. Other steps are the same as above.

For the measurement of apoptosis, TUNEL assays were performed with an *In Situ* Cell Death Detection Kit, following the manufacturer's protocol. DNA strand gaps were identified by TUNEL probes 27. At least 500 cells and four high-powered fields were counted per slide.

In order to take a good contrast picture, we were photographed at 20X and 40X objective lens and applied white balance technology.

### 2.10. Statistical analysis

All data are presented as the mean ± SEM and were analyzed using GraphPad Prism 7.0 software. Statistical analysis was based on Student's t test. All experiments were repeated at least three times to ensure reproducibility. * p<0.05 represents a significant difference from the Ctrl. #p < 0.05 represents a significant difference from the OXA group.

## 3. Results

### 3.1. CBS is overexpressed in colorectal cancer tissues

**Figure 1:**
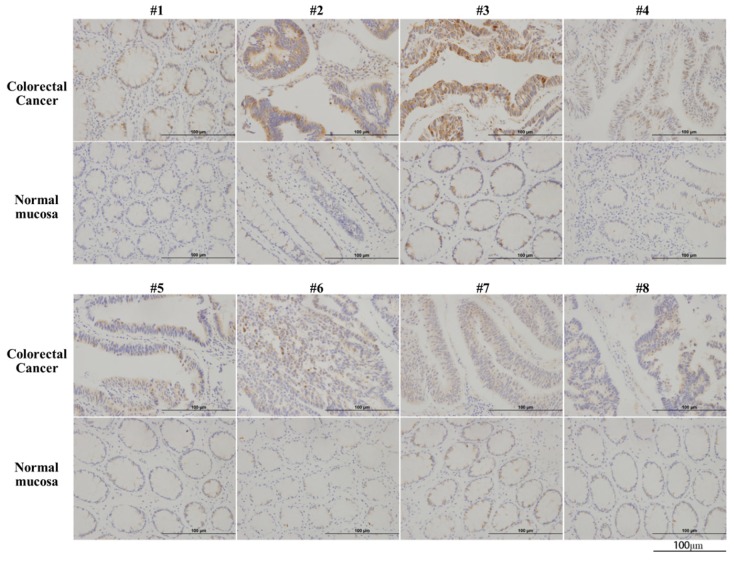
** CBS is overexpressed in human colorectal cancer.** Arabic numerals 1-8 represented pathological specimens from eight different patient sources (scale bar represented 100 µm).

Immunohistochemical analysis of CBS in 11 colorectal cancer tissues and paired normal colon epithelial tissues revealed the significantly upregulated expression of CBS in colorectal cancer tissues (**Fig. 1**).

### 3.2. AOAA inhibited survival of colon cancer cell lines in a concentration-dependent manner

**Figure 2:**
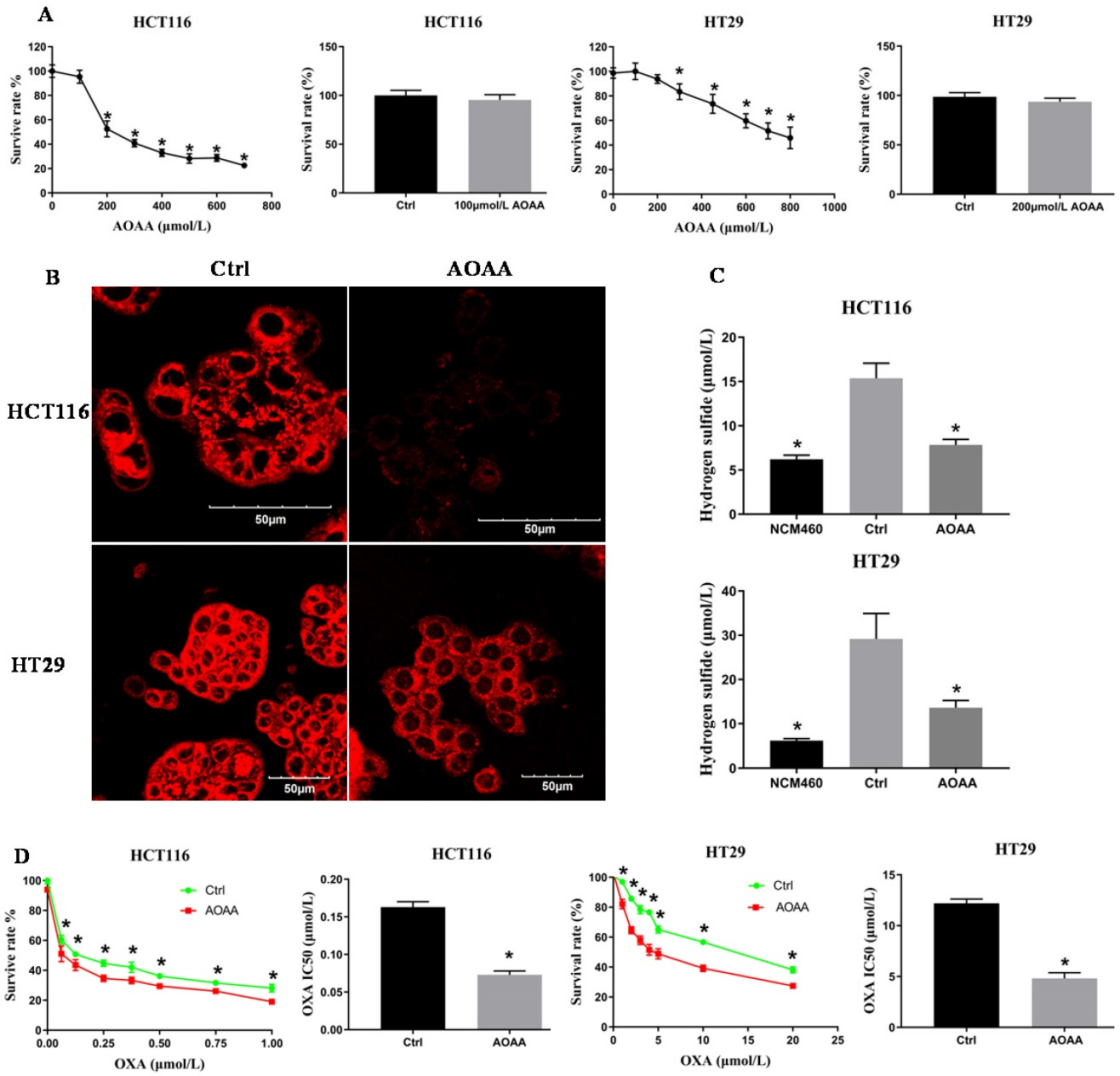
** AOAA inhibited the synthesis of H_2_S and decreased the IC50 values of OXA in HCT116 and HT29 cells. A,** AOAA (100 and 200 µmol/L) had no effect on the proliferation of HCT116 and HT29 cell.** B,** The inhibitory effect of AOAA on the intracellular levels of H_2_S in both cell lines. H_2_S was probed fluorescently. Scale bar represents 50 µm. **C,** AOAA (100 and 200 µmol/L) significantly inhibited intracellular H_2_S synthesis.** D,** AOAA significantly decreased the IC50 values of OXA in both cell lines (*p < 0.05 vs ctrl).

The CCK-8 assay indicated that AOAA inhibited the viability of colon cancer cells in a concentration-dependent manner. AOAA (100 and 200 µmol/L) had no effect on the survival of HCT116 and HT29 cells, respectively (**Fig. 2A**). In the following *in vitro* experiments, AOAA has been using these concentrations in the presence or absence of OXA.

### 3.3. AOAA significantly decreased the intracellular synthesis of H_2_S

The H_2_S content was qualitatively measured by a fluorescent probe. The fluorescence of the AOAA group was significantly weaker than that of the control (**Fig. 2B**). The H_2_S content was quantitatively detected by the electrode. AOAA significantly inhibited intracellular H_2_S synthesis. NCM460 cells, characterized by low expression of H_2_S, served as a negative control (**Fig. 2C**).

### 3.4. AOAA significantly decreased the IC50 values of OXA in HCT116 and HT29 cells

Colon cancer cells were incubated with various concentrations of OXA in the absence or presence of AOAA for 48 hours. Although AOAA (100 and 200 µmol/L) alone had no inhibitory effect on the proliferation of both cell lines, significant decrease in viability was observed in cells treated with OXA in the presence of AOAA compared with cells treated with OXA alone. When OXA was combined with AOAA, the IC50 values of OXA were reduced by 55% and 60.49% in HCT116 and HT29 cells, respectively (**Fig. 2D**).

### 3.5. AOAA significantly exaggerated apoptosis induced by OXA

**Figure 3:**
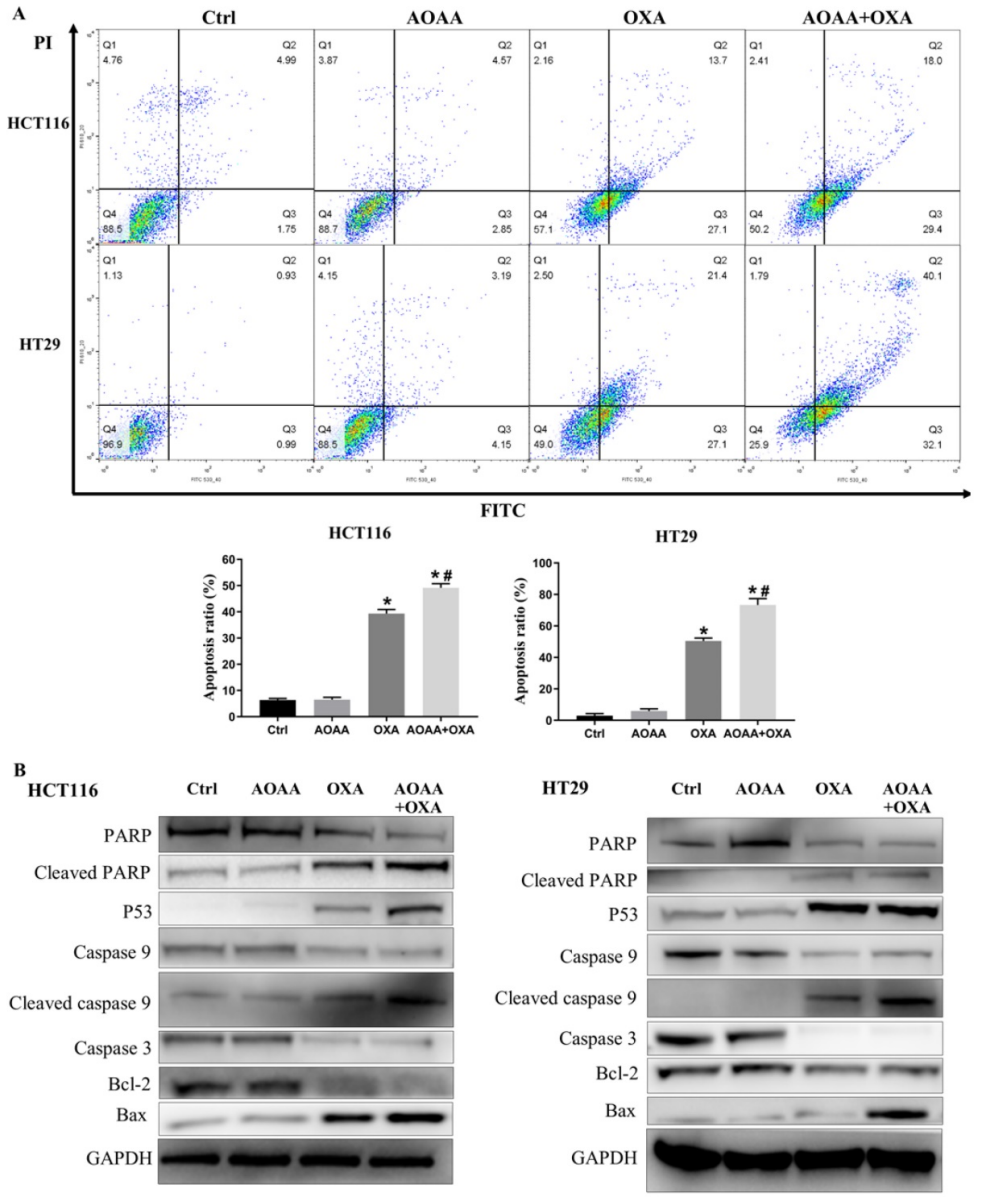
** AOAA exaggerated the apoptosis induced by OXA in both cell lines. A,** Compared with that in OXA group, the proportion of apoptotic cells (early apoptosis + late apoptosis) increased significantly in the AOAA+OXA group. **B,** Co-incubation with AOAA significantly increased the expression of proapoptotic proteins and decreased the expression of antiapoptotic proteins induced by OXA (*p < 0.05 vs ctrl. ^#^ p < 0.05 vs OXA).

Flow cytometry analysis suggested that AOAA significantly increased the apoptosis induced by OXA in both cell lines, featured by increased proportion of cells in the phase of early and late apoptosis (**Fig. 3A**). The OXA-mediated mitochondrial apoptotic pathway is ROS-, P53-mediated and caspase-dependent 18, 19, 21, 28. Interestingly, AOAA upregulates the level of ROS, the expression of P53 protein and inhibits the survival of cancer cells 16, 19. We examined whether OXA combined with AOAA synergized to promote caspase-dependent mitochondrial apoptosis. Western blot analysis showed that the AOAA+OXA group exhibited a significant increase in the expression of cleaved caspase-9, cleaved PARP, Bax and P53 and a significant decrease in the expression of Bcl-2, total caspase-9, total caspase-3 and total caspase-PARP (**Fig. 3B**). Taken together, these data indicated that AOAA and OXA acted synergistically to promote apoptosis through activating apoptotic caspase cascade.

### 3.6. AOAA significantly increased OXA-induced ROS production and decreased intracellular levels of GSH

**Figure 4:**
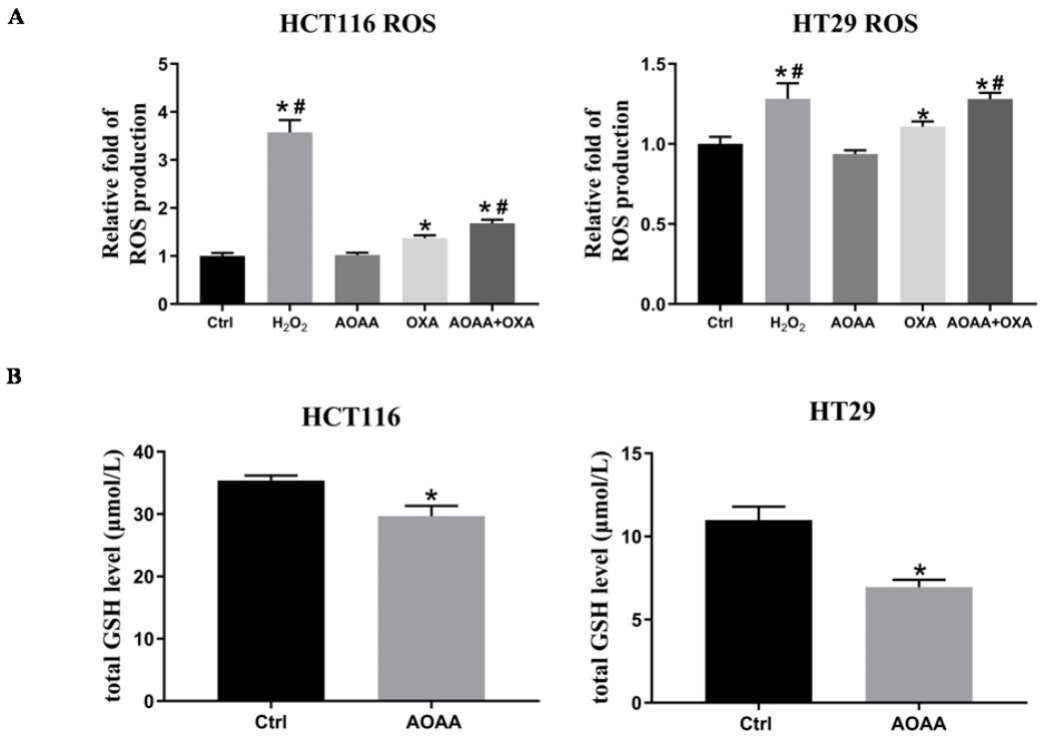
** AOAA significantly increased OXA-induced ROS production and decreased intracellular GSH levels. A,** AOAA significantly increased OXA-induced ROS production. The amount of ROS was calculated with the ratio of DCF-DA/HO signals as described in the section of materials and methods. **B,** AOAA significantly inhibited the synthesis of intracellular GSH (*p < 0.05 vs ctrl. ^#^ p < 0.05 vs OXA).

Previous studies indicated that OXA-induced apoptosis is ROS-dependent 22. Thus, the effect of AOAA on intracellular ROS levels were further investigated. As expected, the results suggested that OXA induced a significant increase in ROS production. AOAA alone did not have a significant effect on ROS levels. What's intriguing is that AOAA significantly exaggerated the increased ROS levels induced by OXA, indicating the pivotal role of ROS in mediating the chemosensitizer role of AOAA (**Fig. 4A**). We also found that AOAA significantly inhibited the synthesis of GSH in HCT116 and HT29 cells, thus impairing detoxification of ROS (**Fig. 4B**). Considering the role of GSH as an important antioxidant, the inhibition of GSH might be one of the mechanisms underlying the effect of AOAA.

### 3.7. AOAA enhanced the chemotherapeutic effect of OXA *in vivo*

**Figure 5:**
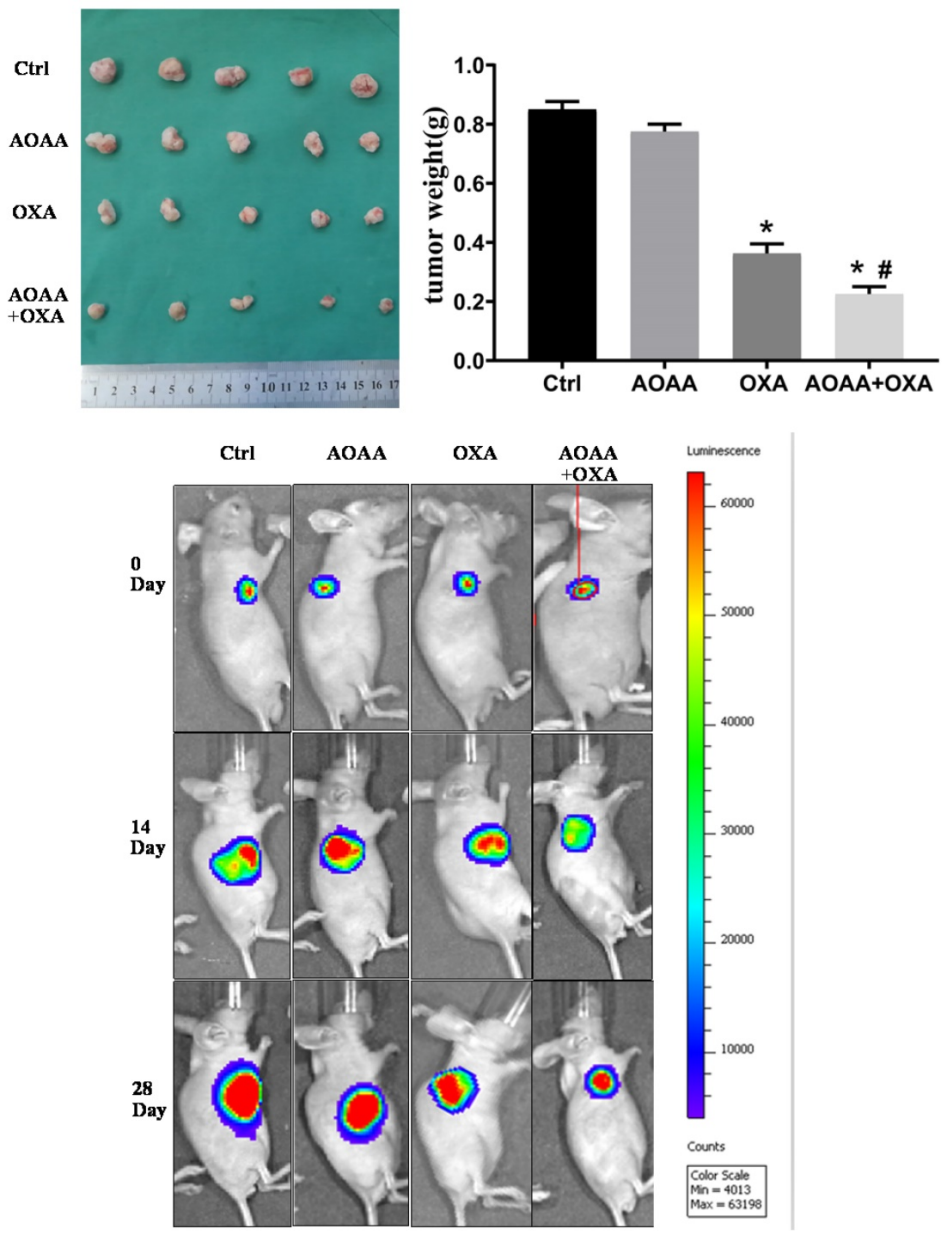
** AOAA sensitizes colon cancer cells to OXA *in vivo*.** Co-treatment with AOAA significantly exaggerated the inhibitory effect of OXA on the tumor growth. (*p < 0.05 vs ctrl. ^#^ p < 0.05 vs OXA).

**Figure 6:**
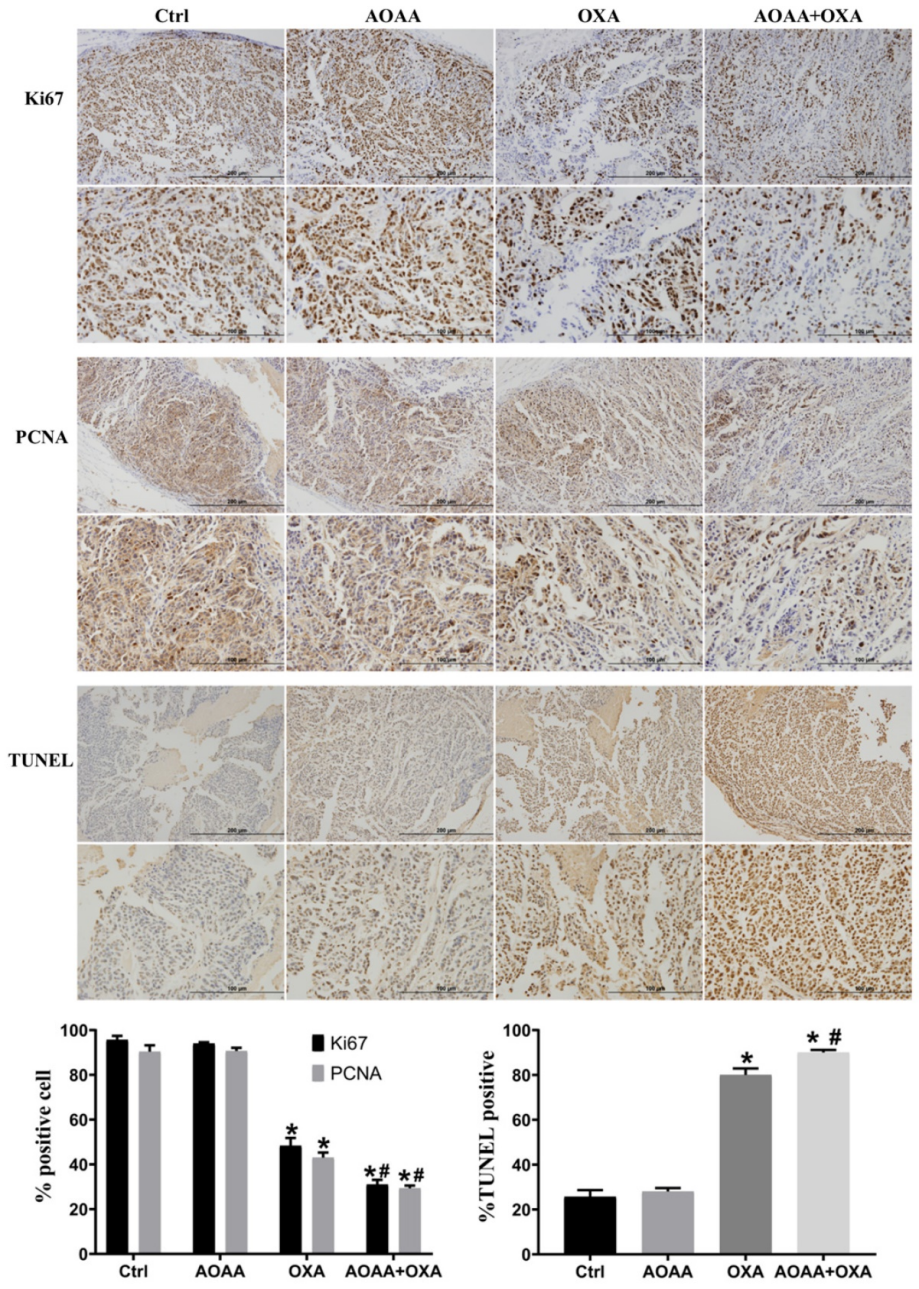
** AOAA sensitizes colon cancer cells to OXA *in vivo*.** Compared with the OXA group, the AOAA+OXA group showed a further decrease in the expression of Ki67 and PCNA. TUNEL assays suggested that co-treatment with AOAA significantly increased the apoptosis induced by OXA (scale bar represented 200 and 100 µm) (*p < 0.05 vs ctrl. ^#^ p < 0.05 vs OXA).

Tumor bearing xenograft mice and *in vivo* imaging systems were used to investigate the effect of AOAA and OXA *in vivo*. The results suggested that OXA alone had a significant inhibitory effect on the tumor growth. AOAA alone had no effect on tumor growth. However, AOAA significantly exaggerated the inhibitory effect of OXA, featured by further decreased tumor weight and volume. *In vivo* imaging of xenograft mice provided visualized evidence, which was also in accordance with the results collected by manual measuring (**Fig. 5**). To evaluate whether OXA combined with AOAA can synergistically amplify apoptosis in xenografts, IHC and TUNEL assays were performed and the results suggested that, the expression of Ki67 and PCNA decreased significantly and the proportion of TUNEL-positive cells increased significantly in tumors treated with both AOAA and OXA (**Fig. 6**). These results suggested that AOAA exaggerated the inhibitory effect of OXA in tumor growth *in vivo* and increased the level of apoptosis induced by OXA.

## 4. Discussion

Increased expression of CBS has been revealed in colorectal cancer tissues and increased tumor-derived H_2_S has been validated to stimulate bioenergetics, cell proliferation, and angiogenesis in colon cancer. Inhibiting synthesis of H_2_S has also been exploited as a promising therapeutic target for cancer and multiple inhibitors of the endogenous synthesis of H_2_S have been developed. However, the effect of inhibiting endogenous production of H_2_S on the chemotherapeutic effect of OXA has not been illustrated. Considering the irreplaceable role of OXA in the chemotherapy of colorectal cancer, investigating the potential role of inhibiting H_2_S as a sensitizer of OXA may shed light on the chemoresistance of colorectal cancer. The current study indicated that inhibition of H_2_S synthesis utilizing AOAA, a classic inhibitor of CBS, significantly sensitized colon cancer cells to OXA both *in vitro* and *in vivo*. AOAA significantly exaggerated the apoptosis induced by OXA and increased the intracellular levels of ROS. Besides, AOAA significantly decreased intracellular levels of GSH, which is an important antioxidant. Together, these results indicated that AOAA could serve as a potential sensitizer of OXA via exaggerating apoptosis in colon cancer cells. Decreased intracellular GSH and the consequently impaired detoxification of ROS might be one of the mechanisms underlying the effect of AOAA.

There are two main apoptotic pathways: the intrinsic mitochondrial apoptosis pathway and the extrinsic death receptor pathway 29. Both modes of apoptosis are involved in chemotherapeutic drugs related cancer cell death. Our results indicated that OXA combined with AOAA induces apoptosis synergistically through the mitochondrial apoptotic pathway. Caspases are essentially a cysteine proteases family, which play a key role in inducing apoptosis by activating downstream substrates 30. Specifically, OXA combined with AOAA activates caspase-3, caspase-9, and PARP. Previous studies have shown that Bcl-2 family proteins also regulate mitochondrial apoptosis pathways 31, 32. We also found that combination treatment significantly upregulated pro-apoptotic Bcl-2 family proteins (Bax), but downregulate anti-apoptotic Bcl-2 family proteins (Bcl-2).

ROS is a collective term that includes O_2_-derived free radicals, such as superoxide anion (O_2_-) and hydroxyl (HO), and O_2_-derived nonradicals, such as H_2_O_2_. ROS are beneficial or harmful to cells in a concentration-dependent manner 29. In cancer cells, ROS are upregulated compared to normal cells 33. However, contrary to tumor-promoting effects, cancer is more sensitive to ROS-induced apoptosis once ROS exceed certain physiological thresholds 17, 29. Therefore, increasing the intracellular ROS concentration is an effective anticancer strategy 20. Previous reports have indicated that OXA-induced apoptosis is mediated by increased intracellular levels of ROS and the consequent mitochondrial apoptosis 3, 17-19. In addition, H_2_S was highly associated with ROS resistance and AOAA upregulated ROS and P53 by inhibiting H_2_S synthesis 7, 12, 16. Our results suggested that, AOAA further increased the intracellular levels of ROS induced by OXA, which might be caused by decreased intracellular GSH levels.

GSH is a major intracellular nonprotein thiol that is one of the main intracellular antioxidants. Studies suggested that GSH could spontaneously (nonenzymatically) inactivates many electrophiles, including ROS and OXA 34, 35. Previous studies suggested that AOAA reduced intracellular cystathionine, a precursor of GSH, thereby decreasing the antioxidant capacity of cells 12, 16, 36. Our results suggested that AOAA synergizes with OXA in inhibiting GSH mediated the depletion of ROS and exaggerating apoptosis.

The acquired resistance to OXA and other chemotherapeutic reagents has been an important risk factor for decreased overall survival in colorectal cancer patients. Increased endogenous levels of H_2_S has been revealed to play an important role in the resistance to 5-FU in colon cancer cells 37. The potential role of H_2_S in the resistance to OXA and the potential role of inhibiting synthesis of H_2_S in restoring sensitivity to OXA in colon cancer cells remains to be illustrated, which will also be the focus of our future work.

In conclusion, this study demonstrates that CBS is overexpressed in human colorectal cancer and AOAA sensitizes colon cancer cells to OXA via increasing apoptosis both *in vitro* and* in vivo*. The inhibition on the intracellular levels of GSH and the consequent decreased detoxification of ROS might be one of the mechanisms underlying the effect of AOAA.

## Abbreviations

H_2_S: Hydrogen sulfide
CRC: Colorectal cancer
CBS: Cystathionine β-synthase
OXA: Oxaliplatin
AOAA: Aminooxyacetic acid
ROS: Reactive oxygen species
GSH: Glutathione
PBS: Phosphate buffer saline
DMEM: Dulbecco's Modified Eagle Medium
ATCC: American tissue culture collection
DMSO: Dimethyl sulfoxide
FBS: Fetal bovine serum
CCK-8: Cell counting kit-8
GAPDH: Glyceraldehyde-3-phosphate dehydrogenase
IC50: Half maximal inhibitory concentration
IHC: Immunohistochemistry
PCNA: Proliferating cell nuclear antigen
TUNEL: Terminal dexynucleotidyl transferase (TdT)-mediated dUTP nick end labeling
